# Cross-cultural adaptation and validation of the Dutch language version of the Pictorial Fear of Activity Scale – Cervical

**DOI:** 10.1186/s12891-020-03724-1

**Published:** 2020-10-28

**Authors:** Maaike Kragting, Lennard Voogt, Koen I. Neijenhuijs, Annelies L. Pool-Goudzwaard, Michel W. Coppieters

**Affiliations:** 1 Department of Physical Therapy, Research Centre for Health Care Innovations, Rotterdam University of Applied Sciences, Rochussenstraat 198, 3015 EK Rotterdam, The Netherlands; 2 Department of Human Movement Sciences, Faculty of Behavioural and Movement Sciences, Amsterdam Movement Sciences, Vrije Universiteit Amsterdam, Amsterdam, The Netherlands; 3 Department of Clinical, Neuro- and Developmental Psychology, Amsterdam Public Health Research Institute, Vrije Universiteit Amsterdam, Amsterdam, The Netherlands; 4 Cancer Center Amsterdam, Amsterdam UMC, Amsterdam, The Netherlands; 5 Somt University of Physiotherapy, Amersfoort, The Netherlands; 6 Menzies Health Insitute Queensland, Griffith University, Brisbane and Gold Coast, Australia

**Keywords:** Fear of movement, Musculoskeletal health, Psychometric properties, Reliability, Validity

## Abstract

**Background:**

The Pictorial Fear of Activity Scale-Cervical (PFActS-C) is a reliable and valid instrument to assess fear of movement in people with whiplash associated disorders. It is not available in Dutch and has not been evaluated in other neck pain populations. This study aimed to systematically translate the PFActS-C into Dutch and evaluate the psychometric properties of this Dutch Language Version (DLV) in people with non-specific neck pain.

**Methods:**

The PFActS-C was translated according to international guidelines. Internal consistency, test-retest reliability, floor and ceiling effects, face validity and construct validity (convergent and discriminant validity by hypotheses testing and structural validity by confirmatory and exploratory factor-analyses) of the PFActS-C-DLV were tested in 125 people with non-specific neck pain.

**Results:**

The PFActS-C-DLV showed good to excellent internal consistency (Cronbach’s alpha: 0.98) and stability over time (ICC: 0.90 [95%CI: 0.82–0.93). Four out of five a priori formulated hypotheses regarding related (convergent validity) and unrelated (discriminant validity) constructs were confirmed. However, the confirmatory factor analysis could not confirm the expected 1-factor solution. Furthermore, the exploratory factor analyses revealed that also a higher factor solution would not lead to a good fit of the model.

**Conclusions:**

The PFActS-C-DLV is a reliable region-specific instrument for people with non-specific neck pain. The construct validity was supported, based on hypotheses testing. However, factor analyses could not confirm a 1-factor solution, so the underlying construct of the PFActS-C-DLV remains unclear. Given the PFActS-C’s photographic format, we believe these findings also have relevance for the original English version.

**Supplementary information:**

**Supplementary information** accompanies this paper at 10.1186/s12891-020-03724-1.

## Background

Neck pain is prevalent and is associated with the fourth largest burden of disease [1]. It has a high rate of transition to recurrent or persistent pain [2]. Psychological factors, such as fear, depression, anxiety and poor coping, are important predictors for the development of pain persistence in people with various musculoskeletal conditions, including neck pain [3, 4]. The importance of assessing and addressing relevant psychological factors in clinical practice is widely accepted [5].

Fear is an important predictor of outcome. Fear is operationalised in several distinct, but closely related constructs, such as fear of movement/reinjury, kinesiophobia, fear of pain and fear avoidance [6]. Fear of movement is described as a specific fear to conduct movements and/or physical activities that are (wrongfully) assumed to cause (re-)injury [6]. Fear of movement may lead to the avoidance of certain activities [7].

Despite limitations, various questionnaires are commonly used to assess fear in people with musculoskeletal pain [6, 8, 9]. The questionnaires focus primarily on cognitions about pain. The items these questionnaires contain, however, are often formulated in rather abstract ways. They sometimes use linguistically difficult constructions, such as double negations (e.g., item 11 in the Tampa Scale of Kinesiophobia [10]) that may be poorly understood by patients [11]. In particular, people with low health literacy (29% in The Netherlands, 47% in Europe [12]) or low-literacy (12% of the people between 15 and 65 in The Netherlands [13]) might be facing challenges when pain is assessed via traditional questionnaires. Furthermore, several questionnaires, initially developed for low back pain or for heterogeneous persistent pain populations, are later applied to other conditions such as neck pain (e.g., the Tampa Scale of Kinesiophobia [10], the Fear Avoidance Beliefs Questionnaire [14] and the Pain Anxiety Symptom Scale [15]. These questionnaires do not specify potentially fearful movements for people with neck pain. This information may be important for interventions aimed at reducing fear of movement. To address these limitations, region-specific questionnaires using pictures instead of verbal statements have been developed. For neck pain, the Pictorial Fear of Activity Scale-Cervical (PFActS-C) was developed [11].

The PFActS-C is a self-report instrument depicting 19 photographs of movements and activities that might elicit fear and can potentially be addressed during therapy, e.g. graded exposure, in people with neck pain and functional disability due to a motor vehicle accident [11]. The photographs depict positions of neck and arms with increasing biomechanical loads. Patients are asked to imagine performing the depicted movement themselves and rate each movement on an eleven point numeric scale, ranging from 0 (no fear at all) to 10 (extreme fear), according to the amount of fear they would experience [11]. The scale can be completed in about 5 min. Internal consistency (Cronbach’s alpha: 0.98), construct validity and stability over time (Intraclass Correlation Coefficient (ICC) = 0.72) of the PFActS-C are good to excellent [11].

The PFActS-C has not yet been translated into other languages and is not yet validated in people with non-specific neck pain. Moreover, no factor analysis has been performed for the PFActS-C-19 item version to evaluate the structure and number of factors the scale measures. Therefore, this study aimed (1) to cross-culturally adapt the PFActS-C into Dutch, and (2) to evaluate its psychometric properties, including a confirmatory and exploratory factor analysis in people with non-specific neck pain. Considering the photographic nature of the PFActS-C and the fact that the text is limited to simple instructions, we believe that the psychometric findings revealed in this study also have relevance for the original version.

## Methods

The PFActS-C was translated into Dutch and cross-culturally adapted to create the PFActS-C Dutch Language Version (PFActS-C-DLV). Following the translation and adaptation, psychometric properties of the PFActS-C-DLV were determined in people with non-specific neck pain in primary care. The study was conducted in accordance with the Declaration of the World Medical Association and was approved by the Scientific and Ethical Review Board (VCWE) of the Faculty of Behavioural and Movement Sciences, Vrije Universiteit Amsterdam, The Netherlands (VCWE-2016-204). All participants provided written informed consent prior to participation.

### Translation and cross-cultural adaptation

Recommendations for the translation and cross-cultural adaptation of self-report measures were followed [16]. In brief, the PFActS-C was translated by two native Dutch speakers (T1 and T2), independently of each other. A preliminary version of the PFActS-C-DLV (T12) was developed in a consensus meeting involving both translators and the principal investigator. Two native English speakers (T3 and T4) who were bilingual (English & Dutch) and blinded to the original English version, independently translated the preliminary PFActS-C-DLV (T12) back into English. Subsequently, the translated versions were submitted to an expert panel of bilingual clinicians, a methodologist, a linguist, the translators and the investigators to identify and address any difficulties, inconsistencies or mistakes in the translation. Adjustments to the photos were not considered, as the panel believed the situation depicted was sufficiently recognisable for people with neck pain.

The revised preliminary version of the PFActS-C-DLV was piloted in a Dutch sample of participants with non-specific neck pain, recruited from a rehabilitation centre and physiotherapy clinics in The Netherlands. This pilot aimed to test whether the PFActS-C-DLV could be completed without assistance, and whether the translated instructions and questionnaire items were unambiguous. Two investigators (MK and LV) evaluated the findings from the pilot study to develop the final version of the PFActS-C-DLV. The reports, and the preliminary and final versions of the PFActS-C-DLV were sent to the original developers of the scale for final approval.

### PFActS-C-DLV scores and psychometric properties

The PFActS-C-DLV was administered in people with non-specific neck pain and per item mean scores, standard deviations (SD) and minimum and maximum scores were assessed.

Reliability (internal consistency, test-retest reliability and measurement error), floor and ceiling effects, face validity and construct validity (convergent, discriminant validity and structural validity by factor-analysis) of the PFActS-C-DLV were assessed according to Consensus-based Standards for the selection of health status Measurement Instruments (COSMIN) guidelines [17, 18]. The statistical methods used are based on the Classical Test Theory (CTT) as developed by Spearman and Cronbach [19]. Statistical analyses were conducted using SPSS (IBM Corp. Released 2015. IBM SPSS Statistics for Windows, Version 23.0. Armonk, NY:IBM Corp) and R studio version 3.5.3 [20] using Lavaan version 0.6–3 [21]. If missing items were present, they were excluded pairwise. *P*-values < 0.05 were considered significant. Data were collected between March 2017 and February 2018.

### Participants

Participants with non-specific neck pain as their primary complaint were recruited from physiotherapy clinics. Participants were screened for eligibility by physiotherapists using the following inclusion criteria: 1) Neck pain Grade I and II [22] (i.e., Grade I: neck pain with no signs of major pathology and no or little interference with daily activities; Grade II: neck pain with no signs of major pathology, but with interference with daily activities that was provoked and/or aggravated by cervical movements, 2) aged between 18 and 80 and 3) being able to read and understand Dutch. Participants were excluded if they had neck pain with neurological signs (i.e., neck pain Grade III) or neck pain with signs of serious pathology (i.e., neck pain Grade IV) [22]. Sample sizes were based on available guidelines: to determine reliability, about 50 participants are required; to determine construct validity, larger samples (> 100) are recommended [23].

#### Reliability

##### Internal consistency

To determine internal consistency, inter-item correlations and item-total correlations were used to determine whether or not an item remained part of a scale. Values of inter-item correlations should be between 0.2 and 0.5 [24]. Corrected item-total correlations > 0.3 contribute to the distinction between patients who are more of less fearful [25]. Cronbach’s alpha was calculated to measure internal consistency of the PFActS-C-DLV. A Cronbach’s alpha with a value > 0.7 is considered acceptable [26].

##### Test-retest reliability and measurement error

For test-retest reliability, the PFActS-C-DLV was administered twice within 1 to 3 weeks. A 7-point Global Rating of Change (GROC) score was used to verify whether neck pain had changed during this period (1: Completely recovered; 2: Much improved; 3: Slightly improved; 4: Not changed; 5: Slightly worsened; 6: Much worsened; 7: Worse than ever [27]). Only data from patients with a GROC score of 3, 4 or 5 (i.e., little or no change in the clinical presentation [28]) were used for test-retest reliability of the PFActS-C-DLV. The time period of 1 to 3 weeks was chosen to limit recall bias and to minimise clinical change.

The intraclass correlation coefficient two-way mixed effects model, single measurement, type agreement with 95% confidence interval (95%CI) (ICC(A,1)) [29] was calculated to measure reliability over time (1 to 3 weeks). ICC values > 0.7 were rated positive [30]. In addition, the standard error of measurement (SEM agreement = √(between administration variance + residual variance) and SEM consistency = √(residual variance) [31]) and the Smallest Detectable Change (SDC = 1.96 * √2 * SEM) [32] were calculated.

##### Floor/ceiling effects

Floor/ceiling effects were considered to be present when > 15% of the participants obtained the lowest or highest possible scores [30].

#### Validity

##### Face validity

Face validity is the extent to which the construct ‘fear of movement’ is represented by the various items of the scale [30]. To assess face validity, a qualitative assessment was performed by an expert panel who was informed about the theoretical background of the construct ‘fear of movement’, the purpose of the PFActS-C-DLV and the target population. The information provided was included in a user manual, and was added to the scale, because it did not exist yet. Subsequently, the panel was asked to check whether the instruction and the pictures in the PFActS-C-DLV were sufficiently relevant and comprehensive to measure fear of movement.

##### Construct validity

Construct validity is the extent to which the scores on an instrument are an adequate reflection of the theoretical construct it aims to measure [17]. To assess construct validity, hypotheses testing and factor analyses were performed.

##### Convergent and discriminant validity (hypotheses testing)

Besides the PFActS-C-DLV, four other questionnaires (Tampa Scale for Kinesiophobia (TSK); Fear Avoidance Beliefs Questionnaire (FABQ); Neck Disability Index (NDI); and Numeric Pain Rating Scale (NPRS)) were administered. These questionnaires measure related (TSK and FABQ) and unrelated (NDI and NPRS) constructs. Five hypotheses (see below) were formulated a priori about the relationship between the PFActS-C-DLV and the other four instruments. Furthermore, the known group method was used [30]. This method compares the scores on the PFActS-C-DLV between two groups that differ with respect to the construct being measured.

When formulating these hypotheses it was taken into account that the TSK and FABQ are related, but not completely comparable with the PFActS-C-DLV, because of the differences in underlying constructs (i.e., kinesiophobia, fear avoidance beliefs and fear of movement) [6]. Although the PFActS-C correlated only moderately to poorly with other instruments that measure fear or anxiety (r ranging from 0.37 to 0.40 [11]), a recent study revealed that the NeckPix (which is another Pictionary scale to measure fear of activities in people with neck pain) had a higher correlation (*r* = 0.76) with measures of kinesiophobia [33]. Therefore, correlations between the scales that measure fear were expected to be at least moderate (r > 0.5), and at least 0.1 higher than correlations with scales that measure different constructs such as pain and/or disability.

#### Questionnaires

##### Tampa scale for Kinesiophobia (TSK)

The TSK is a preferred questionnaire to evaluate kinesiophobia [6, 9]. The TSK consists of 17 items, each scored on a 4-point Likert scale [10, 34]. The total score ranges from 17 to 68, with a score >  37 being regarded as indicative for kinesiophobia [34]. The Dutch version of the TSK is reliable and has been validated in people with low back pain and fibromyalgia [35], but has not yet been validated for neck pain. The psychometric properties of the TSK in people with neck pain have been studied in several countries in various neck pain populations. Reliability and validity are moderate to good [36–38].

##### Fear avoidance beliefs questionnaire (FABQ)

The FABQ is a preferred questionnaire to assess beliefs about how work and physical activity affect pain [6, 9]. The FABQ consist of 16 items scored on a 6-point Likert scale. Four items are related to physical activity (FABQ-pa), 7 items to work (FABQ-w) and there are 5 additional items [14]. The internal consistency, test-retest reliability and validity of the FABQ in people with neck pain is acceptable [36, 37, 39]. A Dutch language version is available [40], but has only been validated in people with low back pain.

##### Neck disability index (NDI)

The NDI assesses the level of disability in people with neck pain and consists of 10 items with six response categories (range 0–5, total score range 0–50, with higher scores representing higher disability) [41]. The Dutch language version of the NDI is reliable and responsive in patients with acute neck pain [42].

##### Numeric pain rating scale (NPRS)

The Numeric Pain Rating Scale is a simple tool to measure pain intensity on a 11-point scale, which is applicable in most settings [43]. It has slightly superior measurement properties as compared to other pain scales [44].

##### A-priori hypotheses

1. The PFActS-C-DLV has a moderate to strong correlation (r > 0.5) [45] with the TSK and FABQ, as all scales aim to assess fear (i.e., convergent validity).
2. The PFActS-C-DLV has a higher correlation (correlation coefficient at least 0.1 higher) with the FABQ-Physical Activity subscale (FABQ-pa) than with FABQ-Work (FABQ-w), as fear of movement is expected to be more related to beliefs regarding physical activity than work.
3. The PFActS-C-DLV correlates stronger (correlation coefficient at least a 0.1 higher) with the TSK and the FABQ-pa (i.e., convergent validity) than with the NDI and the Numeric Pain Rating Scale-average (NPRS-av) and maximal (NPRS-max), because the latter instruments focus more on disability and pain than on fear.
4. The PFActS-C-DLV has no significant correlation with age, because these are different constructs (i.e., discriminant validity).
5. The PFActS-C-DLV score is significant higher in participants who reported a car accident in their history than in participants who reported a gradual onset of pain, because catastrophising (a closely related construct to fear [7, 46]) is a more prominent feature in people who experienced a whiplash trauma [47].

Correlations were calculated to test Hypotheses 1, 2, 3 and 4. Prior to these analyses, the assumption of normality was assessed by visual inspection of the histograms, q-q plots, and the box plots of the data. Skewness and Kurtosis and a Kolmogorov-Smirnov test (*p* < 0.001) were also performed. Outcomes of these analyses determined the use of Pearson’s r or Spearman’s rho statistics [48]. If normality assumptions were not met, 95% Bias-Correction and acceleration Confidence Intervals (BCa CI) were calculated, that corrects for bias and skewness in the distribution of bootstrap estimates [49].

To test Hypothesis 5, the means of 2 subgroups were compared, using an independent t-test in case of normal distribution of the data or a Mann-Whitney U test in case of a non-normal distribution [50, 51]. The construct validity was rated good if at least 75% of the hypotheses are confirmed [30].

##### Structural validity

A confirmative factor analysis (CFA) was performed to verify a 1-factor solution for the PFActS-C-DLV. Model fit was evaluated using Lavaan version 0.6–3 [21], in Rstudio [20] by determining the Root Mean Square Error of Approximation (RMSEA), Comparative Fit Index (CFI), Tucker-Lewis fit Index (TLI) and Standardized Root Mean square Residual (SRMR). Values for RMSEA must be close to 0.06 and SRSR must be below 0.08 [52]. For CFI and TLI a cut off value of 0.95 indicates good model fit [52].

In case of an unacceptable fit for the 1-factor solution, exploratory factor analyses (EFA) using oblimin rotation were performed on all 19 items using Lavaan version 0.6–3 in Rstudio [20] to estimate the underlying factors.

## Results

### Translation and cross-cultural adaptation

Thirty-nine participants with non-specific neck pain (23 females, 16 males; mean (SD) age: 42.9 (9.6) years) filled in the preliminary PFActS-C-DLV (see Additional file 1.). Thirty-four participants indicated that the instruction and items of the PFActS-C-DLV were clear. Some comments were made regarding the weight of the suitcase being unclear, the similarities between the pictures, the lack of clarity about whether or not to consider pain in other body regions, and/or how long a position had to be maintained. These remarks were discussed with the original developers of the scale, who advised that each respondent could use their own criterion rather than specifying a weight for the suitcase. Following this advice, we decided to include the explanation “use your own estimate for the weight of the suitcase” in the accompanying user’s manual (see Additional file 2.). The other comments did not result in adaptations of the scale.

We provided the necessary reports to the original developers and they confirmed that all the steps of the translation were followed appropriately.

### PFActS-C-DLV scores and psychometric properties

Fifty-four participants (39 females, 15 males; 49.7 (12.9) years) who were stable in their clinical presentation completed the PFActS-C-DLV twice to determine the test-retest reliability.

For the other reliability items and validity, 133 participants (97 females, 36 males; 47.0 (12.5) years) participated. Eight participants did not complete all items of the questionnaires and their data were excluded from the analyses pairwise. Characteristics of the included participants are listed in Table 1.

**Table 1:** Participant’s characteristics

Variables	People with non-specific neck pain
**Sex** (*N* = 133) (N (%))	
Women	97 / 73%
Men	36 / 27%
**Education level** (*N* = 131) (N (%))	
Low	25 / 19%
Middle	56 / 43%
High	50 / 38%
**Work situation** (*N* = 131) (N (%))	
Not changed because of neck pain	101 / 77%
Work less because of neck pain	12 / 9%
Stopped working	18 / 14%
**Onset** (*N* = 131) (N (%))	
Traumatic	37 / 28%
Non-traumatic	94 / 72%
**History of neck or low back pain** (*N* = 131) (N (%))	
Yes	102 / 78%
No	29 / 22%
**Kinesiophobia** (*N* = 131) (N (%))	
No (≤ 37 on TSK)	94 / 72%
Yes (> 37 on TSK)	36 / 28%
**Avoid pain** (*N* = 131) (N (%))	
No (≤ 14 on FABQ-pa)	100 / 76%
Yes (> 14 on FABQ-pa)	31 / 24%
**Age** (in years) (*N* = 131) (Mean (SD))	47.0 (12.5)
**Duration** of the episode of neck pain (in months) (*N* = 126) (Mdn (IQR))	
Acute (0 – 1 week) (*N* = 0 / 0%)	12 (45)
Sub-acute (1 week - 3 months) (*N* = 35 / 28%)	2 (2)
Chronic (> 3 months) (*N* = 91 / 72%)	24 (76)
**PFActS-C DLV** (*N* = 125) (Mdn (IQR))	
Total score	57 (89)
Total score in people with traumatic onset (*N* = 32)	100 (96)
Total score in people with non-traumatic onset (*N* = 91)	54 (75)
**NPRS** (*N* = 131) (Mdn (IQR))	
Average last week	6 (3)
Maximum last week	7 (2)
**NDI** (*N* = 131) (Mean (SD))	
Total score	15.2 (8.6)
Total score (%)	30.4 (17.2)
**FABQ** (*N* = 131)	
Total score (Mean (SD))	31.6 (19.0)
Score on FABQ-PA (Mean (SD))	10.3 (5.5)
Score on FABQ-W (Mdn (IQR))	14 (15)
**TSK** (*N* = 131) (Mean (SD))	
Total score	33.8 (7.9)

Abbreviations: *N* Number; *%* Percentage; *SD* Standard Deviation; *Mdn* Median; *IQR* Inter Quartile Range; Education level: low = lower vocational education (primary school; VMBO/LBO/MAVO), middle = high school and/or secondary vocational education (MBO/HAVO/VWO), high = higher professional education and/or university (HBO/WO); *PFActS-C DLV* Pictorial Fear of Activity Scale-Cervical Dutch Language Version; *FABQ* Fear Avoidance Beliefs Questionnaire; *pa* Physical activity; *w* Work; *NDI* Neck Disability Index; *NPRS* Numeric Pain Rating Scale;

The average PFActS-C-DLV scores per item (see Table 2) showed that the movements in which the suitcase is held overhead were perceived as the most threatening. Item 14, in which this position is combined with extension of the neck, was experienced as the most fearful movement (mean (SD) = 4.3 (3.2)).

**Table 2:** Descriptive statistics scores PFActS-C DLV (*N* = 125)

Item	Description	Mean (SD)	Range (Min-Max)
Item 1	Arms@Side Flexion	2.29 (2.81)	10 (0–10)
Item 2	Arms@Side Extension	3.09 (2.87)	10 (0–10)
Item 3	Arms@Side Right Lateral Flexion	2.85 (2.58)	8 (0–8)
Item 4	Arms@Side Left Lateral Flexion	2.90 (2.59)	8 (0–8)
Item 5	Arms@Side Right Rotation	3.23 (2.97)	10 (0–10)
Item 6	Arms@Side Left Rotation	3.16 (2.98)	10 (0–10)
Item 7	Arms@Shoulders Flexion	2.81 (2.83)	10 (0–10)
Item 8	Arms@Shoulders Extension	3.55 (2.88)	10 (0–10)
Item 9	Arms@Shoulders Right Lateral Flexion	3.31 (2.82)	9 (0–9)
Item 10	Arms@Shoulders Left Lateral Flexion	3.38 (2.88)	9 (0–9)
Item 11	Arms@Shoulders Right Rotation	3.60 (2.87)	10 (0–10)
Item 12	Arms@Shoulders Left Rotation	3.45 (2.94)	10 (0–10)
Item 13	Arms Overhead Flexion	4.14 (3.11)	10 (0–10)
Item 14	Arms Overhead Extension	4.32 (3.12)	10 (0–10)
Item 15	Arms Overhead Right Lateral Flexion	4.08 (3.13)	10 (0–10)
Item 16	Arms Overhead Left Lateral Flexion	4.04 (3.14)	10 (0–10)
Item 17	Arms Overhead Right Rotation	4.21 (3.12)	10 (0–10)
Item 18	Arms Overhead Left Rotation	4.09 (3.20)	10 (0–10)
Item 19	Unloaded Arms Overhead Flexion	3.25 (3.14)	10 (0–10)

Abbreviations: *PFActS-C-DLV* Pictorial Fear of Activity Scale-Cervical-Dutch Language Version; *SD* Standard Deviation; *Min* Minimum; *Max* Maximum

#### Reliability

##### Internal consistency

Inter-item correlations varied between 0.55 and 0.97, indicating that the different items of the PFActS-C-DLV measure the same construct and that some items could be removed. Corrected item-total correlation varied between 0.72 and 0.94, indicating that all items contribute to the distinction between patients who are fearful and those who are not fearful. Cronbach’s alpha of the PFActS-C-DLV was 0.98, which is excellent.

##### Test-retest reliability

Test-retest reliability of PFActS-C-DLV was good to excellent with ICC agreement = 0.90 [95%CI: 0.82–0.94]. The SEM agreement was 16.0 (see Table 3), with a corresponding SDC of 44.3.

**Table 3:** Test-retest reliability of the PFActS-C DLV in people with non-specific neck pain (*N* = 54)

	Mean (SD) test	Mean (SD) retest	Mean (SD) Change score	ICC Consistency (95% CI)	ICC Agreement (95% CI)	SEM Consistency	SEM Agreement
PFActS-C-DLV	61.9 (49.7)	55.4 (49.2)	−14.3 (17.7)	0.902 (0.837–0.942)	0.896 (0.823–0.939)	15.5	16.0

Abbreviations: *SEM* Standard Error of Measurement; *ICC* Intraclass Correlation Coefficient; *CI* Confidence Interval

##### Floor/ ceiling effects

Twelve per cent of the participants obtained the lowest possible score (minimum 0). The lower percentile score (25%) was 23.5, with a median score of 57 and a mean score of 65.7. No one obtained the maximum (i.e., worst) score of 190.

#### Validity

##### Face validity

The expert panel consisted of a clinical researcher and four clinicians who were not involved in the translation process. They confirmed that the scale was logical and systematic, and that the pictures were meaningful to evaluate ‘fear of movement’. No missing items were mentioned and all items were considered relevant.

### Construct validity

#### Convergent and discriminant validity (hypotheses testing)

There were no violations of the normality assumptions for the TSK, FABQ, FABQ-pa, and NDI, but the normality assumption was violated for the PFActS-C-DLV, FABQ-w, NPRS-av and NPRS-max scores. Therefore, Spearman’s rho was used to test Hypotheses 1 to 4.

Table 4 shows the correlations between the PFActS-C-DLV and the TSK, FABQ, FABQ-pa, FABQ-w, NDI, NPRS-av, NPRS-max and age. Highest correlations were found between the PFACTS-C-DLV and the FABQ-pa (rs = 0.54, BCa CI [0.38–0.67], p (two-tailed) < 0.01, *R*^*2*^ = 0.29 (*N* = 123)). As hypothesised, the correlation with the NPRS-average was > 0.1 smaller (rs = 0.39, BCa CI [0.20–0.55], p (two-tailed) < 0.01, *R*^*2*^ = 0.15 (*N* = 123)). In contrast to our hypothesis, this was not the case for the correlation with the NDI (r_s_ = 0.49, BCa CI [0.32–0.65], p (two-tailed) < 0.01, *R*^*2*^ = .24 (*N* = 123)).

**Table 4:** Hypothesis testing; correlations between questionnaires and know group differences

A priori hypothesis	Scales or Groups (*N* = number of participants)	PFActS-C-DLV (rs), significance level		Hypothesis Confirmed (yes / no)
1. The PFActS-C has a moderate to strong correlation (r > 0.5) with the TSK and FABQ	TSK (*N* = 122) FABQ (*N* = 123)	0.528, *p* < .001 0.516, *p* < .001		yes
2. The PFActS-C correlates stronger (correlation coefficient at least 0.1 higher) with the FABQ-pa than with FABQ-w	FABQ-pa (*N* = 123) FABQ-w (*N* = 123)	0.540, *p* < .001 0.367, *p* < .001		yes
3. The PFActS-C DLV correlates stronger (correlation coefficient at least 0.1 higher) with the TSK Scale and the FABQ-pa than with the NDI, the NPRS-av and the NPRS-max scores	NDI (*N* = 123) NPRS-av (*N* = 123) NPRS-max (*N* = 123)	0.488, *p* < .001 0.389, *p* < .001 0.300, *p* = .001		no
4. The PFActS-C has no significant correlation with age	Age (*N* = 123)	−0.069, *p* = .448 (ns)		yes
		Median, IQR	Sign. Level	
5. The PFActS-C-DLV score is significant higher in participants who reported a (mechanical) trauma in their history than in participants who reported a gradual onset of pain	Gradual onset group (*N* = 88) Mechanical trauma group (*N* = 24)	Mdn = 52.5, IQR = 89 Mdn = 107.0, IQR = 78	*p* = .006	yes

Validity is expressed by Spearman correlations (rs). Abbreviations: *FABQ* Fear Avoidance Beliefs Questionnaire; *pa* Physical activity; *w* Work; *NDI* Neck Disability Index; *NPRS* Numeric Pain Rating Scale; *av*. Average; *max* Maximal; *IQR* Interquartile Range

To test Hypothesis 5, a Mann-Whitney U test was performed because of the non-normality of the PFActS-C-DLV scores in the group with a gradual onset of pain. Participants who had experienced a car accident scored significantly higher (Mdn = 107, IQR = 89) on the PFActS-C-DLV than participants who reported a gradual onset of pain (Mdn = 52.5, IQR = 78) (U = 670.00, *p* = 0.006, *r* = − 0.26).

For construct validity, four of the five (80%) pre-defined hypotheses were supported.

#### Structural validity

Turk et al. (2008) described the PFActS-C as a questionnaire which measures one underlying construct, namely fear of movement [11]. In the present study, the one-factor structure could not be confirmed by a CFA (Chi-square / df = 1702.91 / 152.00 = 11.20, *p* < 0.001). With a CFI score of 0.67, a TLI score of 0.63, a SRMR score of 0.07 and a RMSEA score of 0.29, a one-factor solution had no adequate fit. EFA’s demonstrated that higher factor solutions (e.g., 2 to 5 factors) would also not lead to a good fit of the model (see Table 5). It is worth mentioning that, despite the fact that there was no adequate fit, some patterns were identified. Examination of the rotated factor loadings from 2, 3, 4 and 5 factor EFA’s suggested that the items with the suitcase above the head (items 13 to 18) reflect the same construct (see Additional files 4, 5, 6 and 7). However, the results of a CFA on these items (13 to 18) remained inconclusive (Chi-square / df = 91.67 / 9.00 = 10.19, *p* < .001, CFI = 0.94, TLI = 0.90, SRMR = 0.02 and RMSEA = 0.27). Furthermore, left and right lateroflexion of the neck (items 3 and 4) and left and right rotation of the neck (items 5 and 6), were consistently paired in the same factor.

**Table 5:** Confirmatory Factor Analysis and Exploratory Factor Analyses

Confirmatory Factor Analysis									
Factor Solution (*N* = 125)	Null Model Chi-square (X^**2**^Bartlett)	Df	Implied Model Chi-square (X^**2**^goodness of fit)	Df	NFI	CFI	SRMR	TLI	RMSEA
**One**	4896.673	171	1702.905	152	0.650	0.670	0.065	0.629	0.286
**Exploratory Factor Analyses**									
**Factor Solution** (*N* = 125)	**Null Model Chi-square (X**^**2**^**Bartlett)**	**Df**	**Implied Model Chi-square** **(X**^**2**^**goodness of fit)**	**Df**	**NFI**	**CFI**	**SRMR**	**TLI**	**RMSEA**
**Two**	4896.673	171	1273.143	134	0.739	0.758	0.044	0.691	0.261
**Three**	4896.673	171	906,549	117	0.786	0.803	0.039	0.712	0.251
**Four**	4896.673	171	744.921	101	0.847	0.863	0.030	0.768	0.226
**Five**	In iteration no local minimum was found. Extraction was terminated.								

Abbreviations: *Df* Degrees of Freedom; *NFI* Normed Fit Index; *CFI* Comparative fit index; *TLI* Tucker-Lewis fit Index; *RMSEA* Root mean square error of approximation

## Discussion

Following the successful translation and cross-cultural adaptation of the PFActS-C 19-item version to Dutch, psychometric assessment revealed good to excellent reliability in people with non-specific neck pain. No floor or ceiling effects were observed. The excellent internal consistency and good stability over time of the PFActS-C-DLV is consistent with the English language version [11]. Therefore, the PFActS-C-DLV can be considered as a reliable and easy to apply region-specific questionnaire.

The SDC of the PFActS-C-DLV is 44.3 (or 23% of the maximal change score of 190) to reveal an actual change (i.e., a change that is larger than the measurement error, not necessarily a change that is clinically meaningful [31]). A possible explanation for this relatively large SDC could be that the ‘slightly improved’ (GROC = 3) and ‘slightly worsened’ (GROC = 5) cases were included in the ‘no change in the clinical presentation’ group. However, a sensitivity analysis on only the people with ‘no change in clinical presentation’ (*N* = 21, GROC = 4) demonstrated a nearly identical SDC (43.9; with comparable other psychometric properties: ICCagreement = 0.91; ICCconsistency = 0.91; SEMagreement = 15.84). Similar size SDC scores are reported for other Dutch language versions of health questionnaires used in people with neck pain, such as the NDI (SDC = 10.5 or 21%; range 0–50) [53]. Reviews of the measurement properties of neck pain related questionnaires (multiple languages versions) report a lack of data concerning SDC values [54] or a large variability of SDC values [55, 56]; for example the SDC scores of the NDI range from 1.7 to 23.3 points on a 50 point scale [56].

Convergent and discriminant validity of the PFActS-C-DLV were good, as 4 out of 5 a priori formulated hypotheses were confirmed. Therefore, construct validity was rated positive, which is consistent with the English-language version [11]. Correlations between the PFActS-C-DLV and NDI (*r* = 0.488) were higher than expected. Even higher correlations between fear and disability were found by Turk (*r* = 0.56) and Monticone (*r* = 0.52) [11, 33]. It could be that disability and/or pain and fear of movement are closely related constructs or that the central question ‘how afraid or fearful would you be to perform the activity shown on this picture?’ was not clear enough. Some participants commented after the measurements that it was difficult to distinguish ‘fearful’ from ‘painful’ or that they just would not perform this movement because of the negative consequences (disability). This may indicate that the PFActS-C-DLV is multidimensional and that the constructs ‘disability’, ‘pain’ and ‘fear of movement’ are overlapping constructs.

Despite the good construct validity with other questionnaires measuring fear, the confirmatory factor analysis could not confirm that the PFActS-C-DLV assesses a single factor as underlying construct, namely fear of movement. Since in the CFA a single factor solution did not provide a good fit of the model, an EFA was performed. However, 2 to 5 factor solutions did not lead to a good fit of the model either. Therefore, the underlying constructs of the PFActS-C-DLV remain elusive and seem to consist of several constructs.

With regard to the structural validity of the PFActS-C-DLV (19 items), an equivalent comparison could not be made with the English language version [11]. In this original study, an EFA was performed (no CFA) on an earlier 41-item version of the PFActS-C in people who had been involved in a motor vehicle accident. Initially, they identified a 5-factor solution [11], but based on further examination of eigenvalues and factor loadings they stated that a single factor solution was the most “parsimonious” solution for the PFActS-C. However, this statement may have to be questioned based on the outcome of the CFA in the present study and the fact that both language versions of the PFActS-C are rather similar due to the limited verbal instruction and the use of identical pictures. Another point to consider is the difference in participants between the original study (people with traumatic neck pain (WAD) with a (mean (SD)) TSK score of 40.2 (5.8) in the moderate to severely group and 35.4 (6.2) in the mild group [11]) and the present study (people with non-specific neck pain with a TSK score of 33.8 (7.9)). Differences in sample characteristics may influence the identified factor structure. However, as the PFActS-C is not exclusively intended for people with traumatic neck pain, we believe that our sample reflected the target population for this questionnaire.

A second explanation for not identifying a clear single factor solution is that although a generic construct might be superior, certain items of the scale may impact differently on different subgroups. For example, fear might be related to a certain direction of movement, i.e. if the neck pain is located on the right-hand side, someone may be afraid only for movements in this direction. This theory would fit with the findings from the EFA’s and the finding of high internal consistency, while the questionnaire does not appear to be unidimensional. Investigations into measurement invariance may shed light upon this possibility. Furthermore, if specific items are related to fear for a certain direction of movement, Item Response Theory models may prove of interest. Finally, it could be that, as described above, multiple constructs such as pain, disability and fear overlap each other.

More research is needed to reveal which underlying constructs are part of the PFActS-C-DLV and to see which items rely on which factor in a specific population. For this reason, it would be valuable to assess neck pain related data (such as the location of the pain, provocative movements and onset of pain (traumatic or non-traumatic) more specifically, so subgroup analyses can be made.

## Conclusions

This study shows us that the PFActS-C-DLV is a reliable region-specific instrument, which is easy to apply in people with neck pain in primary care settings. The construct validity was supported, based on hypotheses testing. However, factor analyses could not confirm a one factor solution, so the underlying construct of the PFActS-C-DLV remains unclear. Therefore, we have to remain cautious with the interpretation of the sum score of the PFActS-C-DLV.

## Supplementary information

**Additional file 1.** PFActS-C-DLV (including photographs used in the PFActS-C).

**Additional file 2.** Manual PFActS-C-DLV.

**Additional file 3.** Description of Items PFActS-C.

**Additional file 4.** Rotated factor loadings of the exploratory 2-factor analysis using oblimin rotation.

**Additional file 5.** Rotated factor loadings of the exploratory 3-factor analysis using oblimin rotation.

**Additional file 6.** Rotated factor loadings of the exploratory 4-factor analysis using oblimin rotation.

**Additional file 7.** Rotated factor loadings of the exploratory 5-factor analysis using oblimin rotation.

**Additional file 8.** Data PFActS-C-DLV.

**Additional file 9.** Data translation and cross-cultural adaptation.

## Data Availability

All data generated or analysed during this study are included in this published article and its supplementary information files.

## Abbreviations

BCaCI: Bias-correction and acceleration confidence intervals
CFA: Confirmatory factor analysis
CFI: Comparative fit index
COSMIN: Consensus-based standards for the selection of health status measurement instruments
CTT: Classical test theory
EFA: Exploratory factor analyses
FABQ: Fear avoidance beliefs questionnaire
FABQ-pa: Fear avoidance beliefs questionnaire physical activity subscale
FABQ-w: Fear avoidance beliefs questionnaire work subscale
GROC: Global rating of change
ICC: Intraclass correlation coefficient
NDI: Neck disability scale
NPRS: Numeric pain rating scale
NPRS-av: Numeric pain rating scale average
NPRS-max: Numeric pain rating scale maximal
PFActS-C: Pictorial fear of activity scale cervical
PFActS-C-DLV: Pictorial fear of activity scale cervical dutch language version
RMSEA: Root mean square error of approximation
SD: Standard deviation
SDC: Smallest detectable change
SEM: Standard error of measurement
SRMR: Standardized root mean square residual
TLI: Tucker-Lewis fit index
TSK: Tampa scale for kinesiophobia
