# Supplementary material for: Cross-cultural adaptation and validation of the Dutch language version of the Pictorial Fear of Activity Scale – Cervical
Source: BMC Musculoskelet Disord. 2020 Oct 28;21:708. doi: 10.1186/s12891-020-03724-1 (PMC7594286; doi:10.1186/s12891-020-03724-1)
Supplement: Supplementary file 1 — Additional file 1. PFActS-C-DLV (including photographs used in the PFActS-C). [file 12891_2020_3724_MOESM1_ESM.docx]

**Additional file 1. PFActS-C-DLV**

**Pictorial Fear of Activity Scale (Dutch language version)**

*Fotoreeks voor het meten van beweging gerelateerde angst bij mensen met nekpijn*

Instructie

Veel mensen met nekpijn raken bezorgd of worden bang om bepaalde activiteiten uit te voeren. De gedachte dat het uitvoeren van deze activiteiten de pijn of het letsel zal verergeren, maakt mensen soms angstig. Wij laten u een reeks foto’s zien van verschillende fysieke activiteiten. Beeld u zich in dat u zelf deze beweging uitvoert. Geef vervolgens bij iedere foto aan hoeveel angst u hierbij ervaart.

Voorzie elke foto van een score van 0 t/m 10 om aan te geven hoeveel angst u ervaart indien u deze activiteit zou uitvoeren; 0 betekent helemaal geen angst en 10 betekent extreme angst. Elke foto heeft een nummer. Dit nummer komt overeen met de nummers op de onderstaande lijst. Slaat u bij het beoordelen alstublieft geen foto’s over.

Geef met behulp van onderstaande schaal aan, hoe bezorgd, angstig of bang u zou worden, wanneer u de getoonde bewegingen uit zou voeren. Houdt hierbij rekening met uw nekpijn.

| Schaal: 0 1 2 3 4 5 6 7 8 9 10   \| Helemaal  geen angst \|  \| Extreme angst \| \| --- \| --- \| --- \| |
| --- | --- | --- | --- |

Nummer foto:

| 1._______ | 5._______ | 9.______ | 13.______ | 17.______ |  |
| --- | --- | --- | --- | --- | --- |
| 2._______ | 6._______ | 10.______ | 14.______ | 18.______ |  |
| 3._______ | 7.______ | 11.______ | 15.______ | 19.______ |  |
| 4._______ | 8.______ | 12.______ | 16.______ |  |  |

**Additional Figure 1**


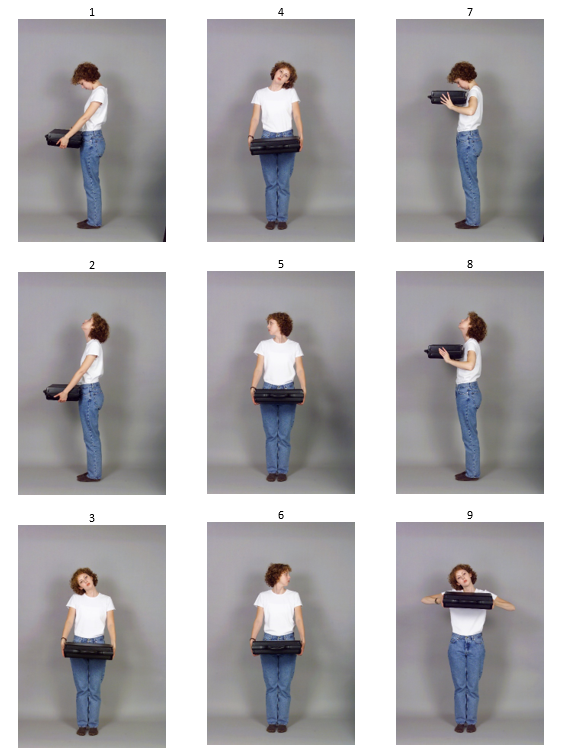


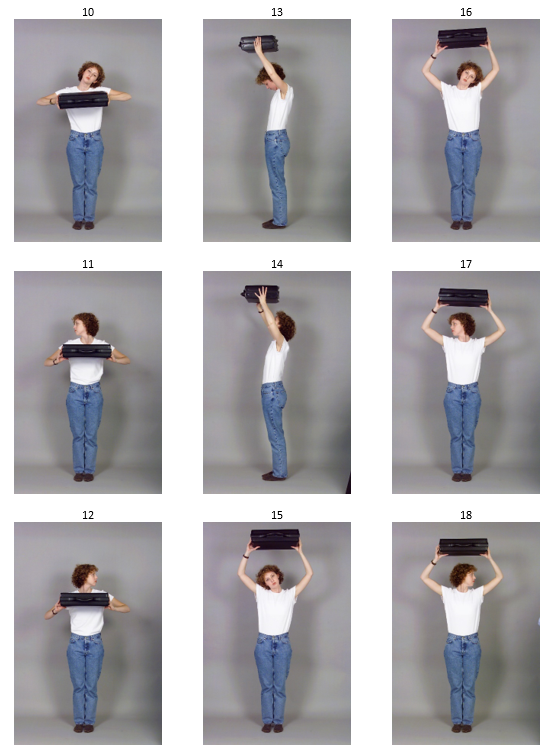


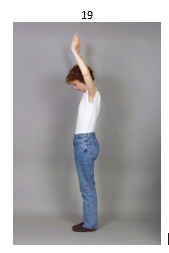


**Additional Fig. 1:** The photographs as used in the original PFActS-C. (The images were obtained from Prof DC Turk, and are reproduced with his permission (personal written communication with Prof DC Turk, 5-2-2020).
