# Supplementary material for: Cross-cultural adaptation and validation of the Dutch language version of the Pictorial Fear of Activity Scale – Cervical
Source: BMC Musculoskelet Disord. 2020 Oct 28;21:708. doi: 10.1186/s12891-020-03724-1 (PMC7594286; doi:10.1186/s12891-020-03724-1)
Supplement: Supplementary file 2 — Additional file 2. Manual PFActS-C-DLV. [file 12891_2020_3724_MOESM2_ESM.docx]

**Additional file 2**. Manual PFActS-C-DLV

Purpose of measurement: To assess fear of movement in patients with non-specific neck pain

Construct of measurement: Fear of movement is described as a specific fear of movement and physical activity that is (wrongfully) assumed to cause reinjury (6). Although we know from the literature that fear of movement, fear of pain, kinesiophobia and fear avoidance beliefs are different constructs (6, 57) the Fear Avoidance Model (FAM) (7, 46) shows us these constructs are closely related to each other. The Pictorial Fear of Activity Scale (PFActS-C), developed by Turk et al. (11)is a specific instrument to assess movement related fear in patients with cervical pain. It uses pictures depicting movements with various specific biomechanical loads, such as neck movements and arm positions. 2 Factors are varied systematically in the 19-items version of this scale, being direction of Movement (flexion, extension, rotation, lateroflexion), Arm Position (At sides, At shoulder height, Overhead). The PFActS-C DLV provides clinicians and researchers specific information about which movements and activities are affected by fear of movement in patients with non- specific neck pain and which can be practiced during (graded exposure) therapy.

Target population: Patients with neck pain grade I/II (i.e. neck pain with no signs of major pathology and no or little interference with daily activities (I) or neck pain with no signs of major pathology, but interference with daily activities (II) (22).

Instructions: The scale is self-explanatory and can be filled in in the waiting room. Patients are asked to imagine themselves performing the same movement as is shown in the 19 pictures and then rate each picture on a 0-10-point numeric scale in which 0 = no fear at all and 10 = extreme fear.

Assistance (when necessary): Sometimes there are questions about the weight of the suitcase: in this case you can tell patients that they can use their own estimate for the weight of the suitcase

Time to complete: 5 minutes
