# Supplementary material for: Cross-cultural adaptation and validation of the Dutch language version of the Pictorial Fear of Activity Scale – Cervical
Source: BMC Musculoskelet Disord. 2020 Oct 28;21:708. doi: 10.1186/s12891-020-03724-1 (PMC7594286; doi:10.1186/s12891-020-03724-1)
Supplement: Supplementary file 3 — Additional file 3. Description of Items PFActS-C. [file 12891_2020_3724_MOESM3_ESM.docx]

**Additional file 3**. Description of Items PFActS-C

| **Item** | **Description** |
| --- | --- |
| Item 1 | Arms at side - Loaded - Flexion |
| Item 2 | Arms at side - Loaded - Extension |
| Item 3 | Arms at side - Loaded - Right lateral bending |
| Item 4 | Arms at side - Loaded - Left lateral bending |
| Item 5 | Arms at side - Loaded - Right Rotation |
| Item 6 | Arms at side - Loaded - Left Rotation |
| Item 7 | Arms at shoulders - Loaded - Flexion |
| Item 8 | Arms at shoulders - Loaded - Extension |
| Item 9 | Arms at shoulders - Loaded - Right lateral bending |
| Item 10 | Arms at shoulders - Loaded - Left lateral bending |
| Item 11 | Arms at shoulders - Loaded - Right Rotation |
| Item 12 | Arms at shoulders - Loaded - Left Rotation |
| Item 13 | Arms overhead - Loaded - Flexion |
| Item 14 | Arms overhead - Loaded - Extension |
| Item 15 | Arms overhead - Loaded - Right lateral bending |
| Item 16 | Arms overhead - Loaded - Left lateral bending |
| Item 17 | Arms overhead - Loaded - Right Rotation |
| Item 18 | Arms overhead- Loaded - Left Rotation |
| Item 19 | Arms overhead - Unloaded - Flexion |

Abbreviations: PFActS-C; Pictorial Fear of Activity Scale-Cervical
