# Supplementary material for: Cross-cultural adaptation and validation of the Dutch language version of the Pictorial Fear of Activity Scale – Cervical
Source: BMC Musculoskelet Disord. 2020 Oct 28;21:708. doi: 10.1186/s12891-020-03724-1 (PMC7594286; doi:10.1186/s12891-020-03724-1)
Supplement: Supplementary file 4 — Additional file 4. Rotated factor loadings of the exploratory 2-factor analysis using oblimin rotation. [file 12891_2020_3724_MOESM4_ESM.docx]

**Additional file 4**. Rotated factor loadings of the exploratory 2-factor analysis using oblimin rotation

| **Item** | **Factor 1** | **Factor 2** |
| --- | --- | --- |
| Item 15 | 0.981 | -0.162 |
| Item 17 | 0.980 | -0.167 |
| Item 18 | 0.980 | -0.155 |
| Item 16 | 0.975 | -0.206 |
| Item 13 | 0.948 | -0.257 |
| Item 14 | 0.923 | -0.265 |
| Item 11 | 0.923 | 0.112 |
| Item 12 | 0.897 | 0.159 |
| Item 19 | 0.874 | -0.123 |
| Item 10 | 0.865 | 0.177 |
| Item 6 | 0.846 | 0.339 |
| Item 9 | 0.839 | 0.193 |
| Item 5 | 0.834 | 0.327 |
| Item 4 | 0.806 | 0.353 |
| Item 8 | 0.803 |  |
| Item 3 | 0.777 | 0.363 |
| Item 7 | 0.763 |  |
| Item 1 | 0.678 |  |
| Item 2 | 0.676 | 0.106 |

Abbreviations: PFActS-C-DLV; Pictorial Fear of Activity Scale-Cervical-Dutch Language Version
