# Supplementary material for: Cross-cultural adaptation and validation of the Dutch language version of the Pictorial Fear of Activity Scale – Cervical
Source: BMC Musculoskelet Disord. 2020 Oct 28;21:708. doi: 10.1186/s12891-020-03724-1 (PMC7594286; doi:10.1186/s12891-020-03724-1)
Supplement: Supplementary file 5 — Additional file 5. Rotated factor loadings of the exploratory 3-factor analysis using oblimin rotation. [file 12891_2020_3724_MOESM5_ESM.docx]

**Additional file 5**. Rotated factor loadings of the exploratory 3-factor analysis using oblimin rotation

| **Item** | **Factor 1** | **Factor 2** | **Factor 3** |
| --- | --- | --- | --- |
| Item 3 | 0.203 | -0.933 |  |
| Item 4 |  | -0.975 |  |
| Item 6 | -0.282 | -0.898 |  |
| Item 5 | -0.141 | -0.880 |  |
| Item 9 |  | -0.621 | 0.313 |
| Item 10 |  | -0.599 | 0.346 |
| Item 12 | -0.262 | -0.539 | 0.378 |
| Item 2 |  | -0.510 | 0.246 |
| Item 1 | 0.181 | -0.458 | 0.316 |
| Item 13 |  |  | 1.008 |
| Item 14 |  |  | 0.986 |
| Item 16 |  |  | 0.961 |
| Item 17 | -0.128 |  | 0.926 |
| Item 18 | -0.218 |  | 0.907 |
| Item 15 |  |  | 0.905 |
| Item 19 |  | -0.152 | 0.754 |
| Item 7 | 0.138 | -0.287 | 0.536 |
| Item 8 |  | -0.339 | 0.508 |
| Item 11 |  | -0.476 | 0.487 |

Abbreviations: PFActS-C-DLV; Pictorial Fear of Activity Scale-Cervical-Dutch Language Version Table 9. Rotated factor loadings of the exploratory 4-factor analysis using oblimin rotation
