# Supplementary material for: Cross-cultural adaptation and validation of the Dutch language version of the Pictorial Fear of Activity Scale – Cervical
Source: BMC Musculoskelet Disord. 2020 Oct 28;21:708. doi: 10.1186/s12891-020-03724-1 (PMC7594286; doi:10.1186/s12891-020-03724-1)
Supplement: Supplementary file 6 — Additional file 6. Rotated factor loadings of the exploratory 4-factor analysis using oblimin rotation. [file 12891_2020_3724_MOESM6_ESM.docx]

**Additional file 6**. Rotated factor loadings of the exploratory 4-factor analysis using oblimin rotation

| **Item** | **Factor 1** | **Factor 2** | **Factor 3** | **Factor 4** |
| --- | --- | --- | --- | --- |
| Item 13 | 1.001 |  |  |  |
| Item 14 | 0.998 |  |  |  |
| Item 16 | 0.890 | -0.170 |  |  |
| Item 17 | 0.883 |  | -0.120 | 0.200 |
| Item 18 | 0.835 |  | -0.215 | 0.162 |
| Item 15 | 0.829 | -0.177 |  |  |
| Item 19 | 0.797 |  | 0.134 | 0.108 |
| Item 7 | 0.532 | -0.135 | 0.190 | 0.141 |
| Item 8 | 0.508 | -0.226 |  | 0.132 |
| Item 1 | 0.446 |  | 0.295 | 0.312 |
| Item 2 | 0.419 |  | 0.206 | 0.342 |
| Item 11 | 0.396 | -0.261 |  | 0.391 |
| Item 9 |  | -0.906 |  |  |
| Item 10 | 0.105 | -0.890 |  |  |
| Item 12 | 0.219 | -0.502 | -0.259 | 0.335 |
| Item 4 |  | -0.471 | 0.164 | 0.463 |
| Item 5 | 0.159 |  |  | 0.854 |
| Item 6 |  | -0.259 | -0.128 | 0.731 |
| Item 3 |  | -0.376 | 0.322 | 0.515 |

Abbreviations: PFActS-C-DLV; Pictorial Fear of Activity Scale-Cervical-Dutch Language Version
