# Supplementary material for: Cross-cultural adaptation and validation of the Dutch language version of the Pictorial Fear of Activity Scale – Cervical
Source: BMC Musculoskelet Disord. 2020 Oct 28;21:708. doi: 10.1186/s12891-020-03724-1 (PMC7594286; doi:10.1186/s12891-020-03724-1)
Supplement: Supplementary file 7 — Additional file 7. Rotated factor loadings of the exploratory 5-factor analysis using oblimin rotation. [file 12891_2020_3724_MOESM7_ESM.docx]

**Additional file 7**. Rotated factor loadings of the exploratory 5-factor analysis using oblimin rotation

| **Item** | **Factor 1** | **Factor 2** | **Factor 3** | **Factor 4** | **Factor 5** |
| --- | --- | --- | --- | --- | --- |
| Item 6 | 0.820 |  | -0.148 |  |  |
| Item 5 | 0.556 | 0.448 |  |  | -0.138 |
| Item 12 | 0.442 |  | -0.442 | 0.165 | -0.235 |
| Item 9 |  |  | -0.923 |  |  |
| Item 10 | 0.108 |  | -0.830 |  | -0.119 |
| Item 3 | 0.190 | 0.424 | -0.436 | -0.212 |  |
| Item 4 | 0.412 | 0.130 | -0.425 | -0.252 |  |
| Item 14 |  |  |  | -0.108 | -1.005 |
| Item 13 |  |  |  |  | -0.973 |
| Item 16 |  |  | -0.170 |  | -0.866 |
| Item 17 | 0.109 | 0.134 |  | 0.208 | -0.853 |
| Item 18 | 0.234 |  |  | 0.146 | -0.834 |
| Item 15 |  |  | -0.209 |  | -0.778 |
| Item 19 |  | -0.140 |  |  | -0.753 |
| Item 8 | 0.139 |  | -0.177 | -0.182 | -0.549 |
| Item 7 |  | 0.272 | -0.211 |  | -0.479 |
| Item 2 | 0.297 |  |  | -0.345 | -0.467 |
| Item 1 |  | 0.346 |  | -0.207 | -0.432 |
| Item 11 | 0.173 | 0.278 | -0.336 | 0.223 | -0.365 |

Abbreviations: PFActS-C-DLV; Pictorial Fear of Activity Scale-Cervical-Dutch Language Version
